# Sexual Offending in Asia: A Psycho-Criminological Perspective

**DOI:** 10.5964/sotrap.12693

**Published:** 2023-09-08

**Authors:** Yasuki Yamaguchi

**Affiliations:** 1 Chief, Supervision Division, Rehabilitation Bureau, Ministry of Justice, Tokyo, Japan

*Sexual Offending in Asia: A Psycho-Criminological Perspective* is beneficial for anyone who is eagerly trying to obtain a comprehensive understanding of sexual offences. The book is divided into two parts. The first part provides a theoretical explanation including a wide range of approaches to explaining sexual offending behaviour. The author’s extensive reviews of past and current studies carried out by researchers in different countries effectively guides us to understand the factors and processes leading to sexual offences as well as profiles of sexual offenders. In addition to theories and models of sexual offending in general, this part also introduces theories and models that explain female sexual offenders and sexual homicide offenders. The second part examines the prevalence, nature, offending dynamics, penal codes, intervention and prevention strategies, and case examples of sexual offending from each region of Asia. With thorough analysis of the cultural background in Asia, one typical cultural context based on patriarchy, which leads to gender-based discrimination and violence against women, is unveiled by the author, and he insightfully associates the cultural distinction with sexual offences in Asia. Finally, the author provides recommendations on measures to reduce sexual offending, which include both preventive efforts such as education and awareness-raising to prevent individuals from becoming a victim or an offender, and treatment and rehabilitation to minimize offenders’ risk of recidivism.

This book enables a broad understanding of theories of sexual offending, and sexual offending issues within different Asian contexts. The extensive studies presented in this book led me to reflect upon my profession as a practitioner in the field of community corrections in Japan. In our country, sexual offenders on probation or parole undergo treatment programmes to prevent reoffending. These programmes address each offender’s problematic behaviour and cognitive distortions, based on the relapse prevention model and cognitive-behavioural therapy. The programme was developed with reference to some programmes that have proven effective in western countries, and Japanese practitioners have a degree of trust in the programme, helped in part by the fact that the results of the evaluation of the programme in Japan were to some extent positive.

However, once the programme used in practice is fixed, we tend to become blind to theories other than the one on which it is based and to factors not dealt with by that programme, which might narrow the possibilities for improving interventions for sexual offenders, including programmes. The author’s work brings new perspectives that highlight a variety of sexual offenders’ pathways which might have been ignored. What astonished me in the latter part was the fact that there is a difference in features of sexual offences derived from cultural background between Asian and western countries. Though we might not place great importance on considering the specific cultural context, such as patriarchal societies, this book reminds us that a focus on cultural context can enhance our understanding of sexual offences. I mentioned this above in the context of the practice in Japan, but there is no doubt that I can make similar suggestions for other regions, especially in Asia. This book distinguishes itself from other equivalent ones with an interdisciplinary perspective, incorporating criminology, sociology and cultural anthropology.

I would strongly recommend this book to the following specific readers with a particular reading suggestion for each. Researchers of sexual offending will be able to gain comprehensive knowledge and a deep understanding of their study subject and recognize features of sexual offences specific to Asian countries with a precise manner. For policy makers and practitioners, especially in Asia, consideration of cultural background will open the door to improved sexual offender strategies. The latter part of the book focuses on describing the prevalence and nature of sexual offences in Asian countries, and descriptions of measures to combat sexual offences are mainly about prevention and responses in early stages rather than the treatment of offenders. Thus, the latter part of the book is particularly recommended for readers involved with such areas.

